# Associations Between Mode of Birth and Neuropsychological Development in Children Aged 4 Years: Results from a Birth Cohort Study

**DOI:** 10.1007/s10578-020-01084-4

**Published:** 2020-10-31

**Authors:** Lea Takács, Samuel P. Putnam, Catherine Monk, Hannah G. Dahlen, Charlene Thornton, František Bartoš, Anastasia Topalidou, Lilian L. Peters

**Affiliations:** 1 Department of Psychology, Faculty of Arts, Charles University, Prague, Czech Republic; 2 Department of Obstetrics & Gynecology, Columbia University, New York, NY USA; 3 Department of Psychology, Bowdoin College, Brunswick, ME USA; 4 Department of Obstetrics & Gynecology, and Psychiatry, Columbia University, New York, NY USA; 5 New York State Psychiatric Institute, New York, NY USA; 6 Research in Childbirth and Health Unit, School of Community Health and Midwifery, Faculty of Health and Care, University of Central Lancashire, Preston, UK; 7 Department of General Practice & Elderly Medicine, University Medical Center Groningen, University of Groningen, Groningen, The Netherlands; 8 Amsterdam UMC, Vrije Universiteit Amsterdam, Department of Midwifery Science AVAG, Amsterdam Public Health Research Institute, Amsterdam, The Netherlands; 9 School of Nursing and Midwifery, Western Sydney University, Sydney, Australia

**Keywords:** Cesarean section, Development, Behavior, Temperament, Pre-school

## Abstract

The aim of this prospective longitudinal study was to examine the association between Cesarean section (CS) and child development and behavior. The sample consisted of 256 children who were born at term without serious perinatal pathologies. Their development and behavior was assessed at the age of four using Ages and Stages Questionnaire (ASQ-3), Children’s Behavior Questionnaire and Strength and Difficulties Questionnaire. Multivariate linear regression analyses were conducted to assess the association between CS and child outcomes. CS was associated with better scores in the Problem Solving domain of the ASQ in the whole sample. After stratifying by child sex, the positive association between CS and the Problem Solving domain was significant in boys, while no association was found in girls. Girls were rated less optimally in the Gross Motor domain of the ASQ when born via CS. Mode of birth was not associated with behavioral outcomes.

**Electronic supplementary material:**

The online version of this article (10.1007/s10578-020-01084-4) contains supplementary material, which is available to authorized users.

## Introduction

In the last few decades, rates of Cesarean section (CS) have risen dramatically. In the United States, the CS rate was 32% in 2015, which is 11% higher than in 1996 [1]. In Europe, the CS rate increased from 11.2 to 25% between 1990 and 2014 [2], rising in almost all countries except for Iceland and Finland. The highest CS rate is reported for Latin America and the Caribbean region (40.5%), though there have been significant increases in many Asian countries as well (from 4.4 to 19.5% between 1990 and 2014) [2].

This rise in CS rates is partly associated with factors that are difficult to change, such as increasing maternal age at first birth [3] and maternal obesity [4]. However, there are other factors that may be easier to alter, such as maternal preference, medical models of care, or funding mechanisms that encourage more frequent intervention in birth. Indeed, after adopting a single, blended payment policy for uncomplicated CS and vaginal births, a decline of 0.27 percentage points per quarter in CS rate was observed in Minnesota, USA [5].

CS is often a life-saving intervention, but may result in adverse effects on maternal and child health. Women who underwent CS have been found to be at higher risk of miscarriage, stillbirth, placenta previa, placenta accreta and placental abruption in future pregnancies [6]. Psychological and psychosocial sequelae, such as reduced maternal self-esteem [7], weaker maternal-infant attachment [8] and higher levels of psychiatric symptoms [9] have been reported as well.

In children, health risks associated with CS are well-documented. Existing reviews and meta-analyses concluded that CS is associated with a higher risk of inflammatory bowel disease [10], asthma [6] and respiratory diseases [11], allergy [12], childhood-onset type 1 diabetes mellitus [13], alterations of immune system functioning [14], atopic diseases [15] and overweight and obesity [6]. Effects on children’s psychological development and behavior are less known, although there is emerging evidence suggesting that CS might impact child neurodevelopment due to modified hypothalamic–pituitary–adrenal axis programming [16], delayed and altered gut microbiota colonization [17], epigenetic changes [18] or altered maternal behaviors [19]. The extant literature in this area, however, is inconclusive, as the studies on this topic are scarce and conflicting in results. Some studies have revealed deficits in cognitive domains [20, 21], delayed motor development [22, 23], or a higher risk of mental health problems, such as autism and Attention-Deficit/Hyperactivity Disorder (ADHD) [24, 25] in children who were born by CS. Some studies, though, found no effects of CS on child psychological outcomes [26–29] and some have reported positive outcomes [30, 31]. These discrepancies might be due to different methodology, such as measurement of outcomes (e.g., parental report or direct child assessment), different sample characteristics, type of CS examined (planned for medical reasons or maternal request, emergency CS), or confounders that are controlled for.

Beyond general expectations that CS may impact developmental progression, potentially delaying acquisition of developmental milestones, CS may also result in alterations in child temperament [32]. Temperament refers to a subset of personality traits that appear early in life and are presumed to have biological underpinnings [33, 34]. In children, factor analyses of multiple temperament dimensions suggest that temperament can be organized into three primary components [35]: *Surgency* concerns individual differences in activity level, impulsivity, and pleasure in situations with high stimulus intensity; *Negative Affectivity* involves demonstrations of sadness, fear, anger, frustration, or discomfort, and difficulties soothing; *Effortful Control* refers to children’s capacities to plan, inhibit inappropriate approach responses, maintain attention, and enjoy low-intensity activities. Temperament has frequently been linked to adjustment in young children [36], and the biological sequelae of CS may thus increase risk of behavior problems [37–39]. Early forms of problems are often demarcated as internalizing (emotional problems including depression, anxiety and peer problems) and externalizing (conduct problems involving aggression, hyperactivity and inattention) [40, 41].

The aim of this study was to examine the association between CS and developmental milestones, temperament, and internalizing and externalizing problems in children aged four years, using data from a birth cohort study. We hypothesized that children born via CS to healthy mothers with no pregnancy complications are at higher risk of developmental delays, behavioral problems, and associated temperament characteristics, compared to children born vaginally. Since those associations might be sex-specific [22, 42], we stratified our analyses for child sex.

## Methods

### Procedure and Participants

This study is a longitudinal project investigating perinatal determinants of child development that commenced at five maternity wards located in non-academic hospitals in the Vysočina Region, Czech Republic, between 2013 and 2014. Mother–child pairs were recruited in the third trimester of pregnancy or during their postpartum stay at the maternity unit. Details about the recruitment of the sample and data collection including maternity system in the Czech Republic are described elsewhere [43]. At baseline, the women were asked to complete a questionnaire about their sociodemographic background. Data regarding labor, delivery and neonatal outcomes were extracted from medical records in collaboration with the hospitals. Children were followed up at the age of four. The mothers were asked to complete questionnaires about their child’s behavior and neurodevelopment, their own health and psychological status, and family sociodemographic background.

Data regarding labor and delivery and data about the sociodemographic background were collected from 1190 women. Out of those women, 343 took part in the follow-up study four years postpartum. The following exclusion criteria were applied: participants were excluded based on multiple pregnancy (n = 3), age < 20 or > 40 years (n = 4), gestational age at birth < 37 or > 42 weeks (n = 11), pregnancy complications (e.g., diabetes, hypertension) (n = 38), birth weight < 2500 g (n = 5), Apgar score at 5 min < 8 or hospitalization of the newborn in maternity hospital > 10 days (n = 5), and vaginal instrumental birth (n = 6). Mother–child pairs with missing values on any of the key study variables were also excluded (n = 15). The final sample thus consisted of 256 mothers and their children (see flow chart, Figure S1, Supplementary Material). A comparison of women who were included in the analyses with those who were not showed that women who were older, had higher education levels or had a spouse were more likely to take part in the follow-up study (Table S1, Supplementary Material).

### Exposure Variable: Mode of Birth

The exposure of interest was mode of birth classified as vaginal birth, planned Cesarean section (CS) without a trial of labor, and emergency CS (CS after a trial of labor).

### Children’s Developmental and Behavioral Outcomes at the Age of Four

Child developmental outcomes were assessed by maternal report using the Ages and Stages Questionnaire–Third Edition (ASQ-3) [44]. The ASQ-3 is a parent-completed questionnaire commonly used in clinical and research settings to screen for developmental delays across five domains of child development: Communication, Fine Motor, Gross Motor, Problem Solving, and Personal-Social domain. Parents are required to evaluate whether the child masters a specific skill (‘yes’), is just beginning to master that skill or masters it only occasionally (‘sometimes’), or has not yet acquired that skill (‘not yet’), with a score of 10, 5 or 0, respectively. Each domain contains 6 items. A total score for each subscale is calculated as a sum of the points ranging from 0 to 60. The higher the score, the better the skills and abilities in the given domain. The psychometric characteristics of the ASQ-3 are satisfactory, as reported by Schonhaut et al. [45].

Child temperament was measured using the Very Short Form of the Children’s Behavior Questionnaire (CBQ-VSF) [35]. The CBQ-VSF is a widely used tool to assess young children’s reactivity and regulation; it contains 36 items divided into three subscales: Surgency, Negative Affectivity and Effortful Control. Each subscale consists of 12 items rated on a 7-point scale ranging from 1 to 7. The total score for each subscale represents the mean score of all scale items applicable to the child during the last 6 months. The authors reported satisfactory internal consistency for the CBQ-VSF scales [35].

Child internalizing and externalizing problems were assessed by the Strength and Difficulties Questionnaire (SDQ) [41]. The SDQ is a widely used questionnaire for mental health screening that consists of scales measuring five dimensions (Emotional Symptoms, Conduct Problems, Hyperactivity/Inattention, Peer Relationship Problems, Prosocial Behavior), with each scale containing five items. In this study, we used 20 items of the SDQ divided into the Internalizing and Externalizing Problems subscales [41]. The Internalizing Problems subscale consists of the Emotional Symptoms and Peer Relationship Problems dimensions. The Externalizing Problems subscale covers Conduct Problems and Hyperactivity/Inattention domains. The items are rated on a 3-point scale ranging from 0 to 2 points such that the informant (parent or teacher) evaluates whether the statement is ‘Not True’, ‘Somewhat True’ or ‘Certainly True’. Both Internalizing and Externalizing scores may range from 0 to 20, with the higher scores indicating more severe problems. The SDQ was reported to have satisfactory psychometric properties [46].

### Control Variables

The following potential covariates were determined based on the previous literature: parity, child’s sex, gestational age at birth [21, 22, 30] and induction of labor [47]. In addition, maternal depressive symptoms [48] were included to control for maternal psychological status at the time of child assessment using the Beck Depression Inventory (BDI-II) [49]. The BDI-II is a widely used questionnaire consisting of 21 items that are rated on a 4-point scale ranging from 0 to 3. The total score may range from 0 to 63, with higher scores indicating a higher level of depressive symptoms. In this study, a validated Czech version of the BDI-II showing high reliability (Cronbach’s alpha 0.92) was used [50].

### Statistical Analyses

Descriptive statistics and *chi-square* tests were calculated on the maternal characteristics to assess the differences between women who were included and excluded in the present study. The descriptive statistics were used to report maternal, childbirth, and child characteristics. Bivariate associations of mode of birth with maternal, childbirth and child characteristics were calculated with *chi square* tests, and bivariate associations of birth mode with measurement scores were calculated with Student *t*-tests or ANOVA-tests, where appropriate.

Univariate and multivariate linear regression analyses were conducted to further assess associations between CS and children’s developmental (ASQ subscales) and behavioral (CBQ-VSF and SDQ subscales) outcomes. The requirements to comply with the assumptions of the multivariate linear regression were evaluated. The unadjusted and adjusted associations between CS and children’s outcomes were reported with a Beta coefficient including 95% confidence interval (95% CI). The corresponding percentages of explained variances (R^2^) of all regression models were also calculated. In the multivariate model, the effect of CS was adjusted for artificial hormones, i.e. oxytocin/prostaglandin for induction of labor (yes/no), parity (nulliparous vs. multiparous women), gestational age at birth (continuous variable, weeks of gestation) and maternal depression (continuous BDI-II score). Additionally, the linear regression models were stratified for child sex. The statistical analyses were performed using SPSS Statistics version 23.0 (SPSS Inc., Chicago, IL, USA).

## Results

### Sample Characteristics

A total of 256 women with uncomplicated pregnancy and their children (126 girls, 130 boys) with no perinatal complications were included in the analysis. The mean maternal age at child’s birth was 30.9 (SD = 3.8) years. Over 47.3% (n = 121) of mothers were nulliparous and 91.4% (n = 234) of them were married or cohabiting at child’s age of 4 years. A majority of the children (77.3%, n = 198) were born between 38 and 40 weeks of gestation. The mean Apgar score at 5 min was 9.7 (SD = 0.5) (Tables 1 and 2).

**Table 1:** Maternal and child characteristics; data on labor and mode of birth collected from medical records from five maternity wards of non-academic hospitals located in the Vysočina Region, Czech Republic, between 2013 and 2014

	Total sample	Vaginal birth	Cesarean section
	*n* = 256 100%	*n* = 192 75%	*n* = 64 25%
Maternal characteristics, *n* (%)			
Age			
20–24 years	12 (4.7)	10 (5.2)	2 (3.1)
25–29 years	79 (30.9)	59 (30.7)	20 (31.3)
30–34 years	119 (46.5)	88 (45.8)	31 (48.4)
35–40 years	46 (18)	35 (18.2)	11 (17.2)
Educational level			
Vocational	20 (7.8)	16 (8.3)	4 (6.3)
Secondary	122 (47.7)	93 (48.4)	29 (45.3)
University	111 (43.4)	81 (42.2)	30 (46.9)
Missing	3 (1.2)	2 (1.0)	1 (1.6)
Marital status 4 years postpartum			
Partner/spouse	234 (91.4)	175 (91.1)	59 (92.2)
Single/divorced	20 (7.8)	15 (7.8)	5 (7.8)
Missing	2 (0.8)	2 (1.0)	
Parity			
Nulliparous	121 (47.3)	85 (44.3)	36 (56.3)
Multiparous	135 (52.7)	107 (55.7)	28 (43.8)
Maternal health status			
Serious health problems 0–4 years postpartum^a^	15 (5.9)	11 (5.7)	4 (6.3)
Childbirth characteristics, *n* (%)			
Pain relief			
None	171 (66.8)	171 (89.1)	–
Epidural analgesia	6 (2.3)	6 (3.1)	–
Spinal analgesia	55 (21.5)	–	55 (85.9)
Other analgesia	24 (9.4)	15 (7.8)	9 (14.1)
Artificial hormones for induction of labor			
None	208 (81.2)	157 (81.8)	51 (79.7)
Oxytocin	8 (3.1)	6 (3.1)	2 (3.1)
Prostaglandin	31 (12.1)	23 (12.0)	8 (12.5)
Both artificial hormones	9 (3.5)	6 (3.1)	3 (4.7)
Child characteristics, *n* (%)			
Sex			
Female	126 (49.2)	94 (49.0)	32 (50.0)
Male	130 (50.8)	98 (51.0)	32 (50.0)
Gestational age at birth			
37 weeks	7 (2.7)	6 (3.1)	1 (1.6)
38–40 weeks	198 (77.3)	151 (78.6)	47 (73.4)
41–42 weeks	51 (19.9)	35 (18.2)	16 (25.0)
Birth weight (grams)			
2500–2999	27 (10.5)	17 (8.9)	10 (15.6)
3000–3499	104 (40.6)	78 (40.6)	26 (40.6)
3500–3999	90 (35.2)	72 (37.5)	18 (28.1)
> 4000 g	35 (13.7)	25 (13.0)	10 (15.6)
Length of postpartum hospitalization of the newborn			
3 days	34 (13.3)	34 (17.7)	–
4–5 days	174 (68.0)	132 (68.8)	–
6–7 days	38 (14.8)	16 (8.3)	42 (65.6)
7–9 days	10 (3.9)	10 (5.2)	22 (34.4)

^a^Mothers reported whether they had experienced any serious health problems following child’s birth

**Table 2:** Mean scores on maternal depressive symptoms (Beck Depression Inventory), measures of developmental (Ages and Stages Questionnaire) and behavioral (Children’s Behavior Questionnaire; Strength and Difficulties Questionnaire) outcomes in children, and Apgar score at 5 min

	Sample completed measures *n* (%)	Score total sample Mean (SD)^a^	Score vaginal birth Mean (SD)^a^	Score CS Mean (SD)^a^	Statistical differences between mode of birth groups *p*-value
Maternal outcomes					
Maternal depressive symptoms 4 years postpartum (BDI-II)	229 (89.5)	5.6 (5.9)	5.7 (6.2)	5.4 (4.6)	0.73
Child outcomes					
Apgar score at 5 min	256 (100.0)	9.7 (0.5)	9.6 (0.5)	9.7 (0.5)	0.38
Developmental outcomes					
ASQ Communication	204 (79.7)	54.4 (8.5)	54.1 (9.0)	55.2 (6.9)	0.42
ASQ Fine Motor	208 (81.3)	48.1 (12.7)	48.4 (12.8)	47.1 (12.4)	0.52
ASQ Gross Motor	211 (82.4)	52.7 (9.1)	53.1 (9.0)	51.7 (9.5)	0.34
ASQ Problem Solving	209 (81.6)	52.5 (10.1)	51.6 (11.0)	55.4 (6.2)	0.002
ASQ Personal-Social	205 (80.1)	53.2 (8.5)	53.4 (8.8)	52.4 (7.7)	0.46
Behavioral outcomes					
CBQ-VSF Effortful Control	254 (99.2)	5.4 (0.9)	5.4 (0.9)	5.5 (0.9)	0.44
CBQ-VSF Negative Affectivity	254 (99.2)	3.9 (0.9)	3.9 (0.8)	3.9 (0.9)	0.66
CBQ-VSF Surgency	254 (99.2)	4.4 (0.9)	4.4 (0.9)	4.3 (0.9)	0.61
SDQ Externalizing Problems	243 (94.9)	10.4 (3.1)	10.5 (2.9)	10.0 (3.4)	0.22
SDQ Internalizing Problems	237 (92.6)	6.6 (2.3)	6.6 (2.2)	6.5 (2.5)	0.61

^a^Standard deviation

Vaginal birth occurred in 75.0% (n = 192) of women, whereas 10.9% (n = 28) had a planned Cesarean section (CS) and 14.1% (n = 36) had an emergency CS. Out of the women included in our sample, 15 women (5.9%) who had experienced a previous CS experienced a planned CS (n = 12) or emergency CS (n = 3) at the index birth. Girls and boys did not differ in terms of likelihood of CS (CS birth occurred in 25.4% girls and 24.6% boys), but boys were more likely to be born at a higher gestational age (22.3% boys were born at 41–42 weeks of gestation compared to 17.5% girls). The indications for performing CS were as follows: fetal hypoxia (26.6%, n = 17), failure to progress (23.4%, n = 15), previous CS (18.8%, n = 12), breech presentation (14.1%, n = 9), and reasons unknown (17.2%, n = 11). As the groups of women distinguished by the type of CS were relatively small (28 for planned and 36 for emergency CS) and there were no statistically significant differences between children born via planned and emergency CS on the outcome variables (except for the CBQ-VSF subscale Negative Affectivity where children born via emergency CS had significantly higher mean score than those born via planned CS) (see Table S2, Supplementary materials), we combined the planned and emergency CS groups in the analyses.

In the vaginal birth group, pain relief was provided to 10.9% (n = 21) of the women, a minority of laboring women (18.8%, n = 48) received oxytocin and/or prostaglandin for induction of labor. Out of the women who gave birth vaginally, 47.4% (n = 91) underwent an episiotomy, 28.5% (n = 55) experienced perineal trauma and one woman had severe blood loss (≥ 1000 ml). When comparing the women and children from the vaginal birth group with those from the CS group, no significant differences in their sociodemographic, perinatal, and health characteristics were found, except for the provision of pain relief (p ≤ 0.001) (Table 1). Women who gave birth vaginally did not show any statistically significant differences in depressive symptoms 4 years postpartum compared to those in the CS group (5.7; SD = 6.2 vs. 5.4; SD = 4.6) (Table 2).

### Children’s Developmental and Behavioral Outcomes

Children who were born vaginally did not differ significantly from those born via CS on the mean scores of the ASQ, CBQ-VSF, SDQ subscales, except for the ASQ subscale Problem Solving. Children born by CS showed a significantly higher mean score compared to children who were born vaginally (55.4; SD = 6.2 vs. 51.6; SD = 11.0), suggesting that the CS born children achieved better outcomes in the Problem Solving domain (Table 2).

All multivariate models complied with the assumptions to conduct a linear regression analysis. In the regression analysis, CS was not significantly associated with developmental and behavioral outcomes, except for the Problem Solving domain (ASQ subscale) such that the CS born children were found to have better scores (unadjusted Beta = 3.84; 95% CI 0.66 to 7.02; adjusted Beta = 4.26; 95% CI 1.22 to 7.31) (Table 3). After stratifying by child sex, the association between CS and the Problem Solving domain was significant in boys (adjusted Beta = 6.64; 95% CI 1.00 to 12.28), while no association was found in girls (adjusted Beta = 1.86; 95% CI − 0.67 to 4.40). Girls born via CS were rated less optimally in the Gross Motor domain (ASQ subscale) (adjusted Beta = − 5.26; 95% CI − 9.24 to − 1.09) (Table 3). Mode of birth was not associated with differences in child temperament domains or behavioral problems in the stratified analysis (Table 4).

**Table 3:** Associations between Cesarean section and developmental outcomes (ASQ subscales) calculated for total sample and stratified by child sex

	ASQ Communication		ASQ Fine Motor		ASQ Gross Motor		ASQ Problem Solving		ASQ Personal-Social	
	R^2^	B (95% CI)^a^	R^2^	B (95% CI)	R^2^	B (95% CI)	R^2^	B (95% CI)	R^2^	B (95% CI)
*Univariate models*										
Mode of birth (CS)	≤ 0.01	1.11 (− 1.59 to 3.82)	≤ 0.01	− 1.32(− 5.36 to 2.72)	≤ 0.01	− 1.39 (− 4.23 to 1.45)	0.03	3.84 (0.66 to 7.02)*	≤ 0.01	− 1.02 (− 3.76 to 1.72)
Induction of labor	≤ 0.01	0.41 (− 2.75 to 3.58)	≤ 0.01	− 0.84 (− 5.54 to 3.86)	≤ 0.01	0.09 (− 3.21 to 3.39)	≤ 0.01	0.01 (− 3.74 to 3.77)	≤ 0.01	0.01 (− 3.19 to 3.22)
Parity	0.04	− 3.27 (− 5.59 to − 0.95)*	≤ 0.01	− 1.37 (− 4.85 to 2.11)	≤ 0.01	− 1.53 (− 4.01 to 0.95)	0.02	− 2.61 (− 5.36 to 0.14)	≤ 0.01	0.32 (− 2.04 to 2.68)
Gestational age at birth	≤ 0.01	− 0.73 (− 1.90 to 0.44)	0.02	− 1.87 (− 3.56 to − 0.17)*	≤ 0.01	0.18 (− 1.02 to 1.39)	≤ 0.01	− 0.84 (− 2.18 to 0.51)	≤ 0.01	− 0.20 (− 1.36 to 0.95)
Child sex (boy)	≤ 0.01	− 1.40 (− 3.75 to 0.96)	0.16	− 10.07 (− 13.27 to − 6.88)*	≤ 0.01	0.56 (− 1.92 to 3.04)	0.12	− 6.97 (− 9.57 to − 4.37)*	0.06	− 4.00 (− 6.29 to − 1.71)*
Maternal depressive symptoms (BDI-II)^b^	≤ 0.01	− 0.16 (− 0.36 to 0.05)	≤ 0.01	− 0.14 (− 0.43 to 0.15)	0.02	− 0.23 (− 0.45 to − 0.02)*	0.02	− 0.23 (− 0.47 to − 0.03)*	0.03	− 0.23 (− 0.43 to − 0.03)*
*Multivariate model total sample*										
Mode of birth (CS)	0.06	1.02 (− 1.72 to 3.77)	0.18	− 0.64 (− 4.41 to 3.12)	0.04	− 1.63 (− 4.65 to 1.39)	0.20	4.26 (1.22 to 7.31)*	0.09	− 0.69 (− 3.49 to 2.11)
Induction of labor		1.38 (− 1.99 to 4.75)		1.51 (− 3.04 to 6.06)		0.39 (− 3.26 to 4.04)		2.59 (− 1.09 to 6.28)		1.77 (− 1.68 to 5.22)
Parity		− 3.29 (− 5.73 to − 0.84)*		− 1.71 (− 5.01 to 1.59)		− 1.41 (− 4.09 to 1.27)		− 2.09 (− 4.76 to 0.58)		0.86 (− 1.59 to 3.32)
Gestational age at birth		− 0.90 (− 2.14 to 0.34)		− 1.64 (− 2.27 to 0.00)*		0.51 (− 0.81 to 1.83)		− 1.02 (− 2.33 to 0.29)		− 0.12 (− 1.33 to 1.09)
Child sex (boy)		− 0.53 (− 2.94 to 1.88)		− 9.50 (− 12.76 to − 6.25)*		0.74 (− 1.90 to 3.38)		− 7.13 (− 9.76 to − 4.51)*		− 3.99 (− 6.39 to − 1.59)*
Maternal depressive symptoms (BDI-II)^b^		− 0.12 (− 0.32 to 0.08)		− 0.12 (− 0.39 to 0.15)		− 0.24 (− 0.46 to − 0.02)*		− 0.19 (− 0.41 to 0.03)		− 0.25 (− 0.44 to − 0.05)*
*Multivariate model girls*										
Mode of birth (CS)	0.05	− 0.63 (− 4.32 to 3.06)	0.06	0.00 (− 4.48 to 4.48)	≤ 0.01	− 5.26 (− 9.24 to − 1.09)*	0.06	1.86 (− 0.67 to 4.40)	0.07	− 2.14 (− 5.30 to 1.02)
Induction of labor		2.69 (− 1.92 to 7.29)		− 0.04 (− 5.28 to 5.20)		2.61 (− 2.39 to 7.62)		0.50 (− 2.57 to 3.56)		1.60 (− 2.28 to 5.48)
Parity		− 2.05 (− 5.20 to 1.11)		1.17 (− 2.56 to 4.90)		− 2.55 (− 6.11 to 1.01)		− 0.05 (− 2.19 to 2.08)		− 0.83 (− 3.51 to 1.84)
Gestational age at birth		− 0.64 (− 2.33 to 1.05)		− 1.73 (− 3.68 to 0.23)		− 0.04 (− 1.88 to 1.80)		− 0.37 (− 1.49 to 0.76)		− 0.74 (− 2.16 to 0.68)
Maternal depressive symptoms (BDI-II)^b^		− 0.14 (− 0.40 to 0.11)		− 0.20 (− 0.49 to 0.10)		− 0.23 (− 0.51 to 0.05)		− 0.14 (− 0.31 to 0.03)		− 0.20 (− 0.40 to 0.01)
*Multivariate model boys*										
Mode of birth (CS)	0.11	2.71 (− 1.48 to 6.89)	0.05	− 1.26 (− 7.41 to 4.89)	0.05	1.89 (− 2.51 to 6.29)	0.14	6.64 (1.00 to 12.28)*	0.06	1.03 (− 3.69 to 5.75)
Induction of labor		0.23 (− 4.87 to 5.32)		3.72 (− 4.06 to 11.51)		− 1.70 (− 7.12 to 3.72)		4.59 (− 2.28 to 11.45)		1.81 (− 3.98 to 7.61)
Parity		− 4.74 (− 8.62 to − 0.86)*		− 4.79 (− 10.45 to 0.87)		− 0.20 (− 4.26 to 3.86)		− 4.64 (− 9.79 to 0.51)		2.87 (− 1.42 to 2.51)
Gestational age at birth		− 1.33 (− 3.21 to 0.54)		− 1.56 (− 4.25 to 1.14)		0.95 (− 0.97 to 2.87)		− 1.52 (− 3.89 to 0.85)		0.55 (− 1.42 to 2.51)
Maternal depressive symptoms (BDI-II)^b^		− 0.12 (− 0.46 to 0.22)		− 0.03 (− 0.51 to 0.46)		− 0.32 (− 0.67 to 0.03)		− 0.28 (− 0.72 to 0.15)		− 0.34 (− 0.70 to 0.02)

*p ≤ 0.05

^a^Beta coefficient in the linear regression analyses

^b^Continuous score of the Beck Depression Inventory

**Table 4:** Associations between Cesarean section and child behavioral outcomes (CBQ-VSF and SDQ subscales) calculated for total sample and stratified by child sex

	CBQ-VSF Effortful Control		CBQ-VSF Negative Affectivity		CBQ-VSF Surgency		SDQ Externalizing Problems		SDQ Internalizing Problems	
	R^2^	B (95% CI)^a^	R^2^	B (95% CI)	R^2^	B (95% CI)	R^2^	B (95% CI)	R^2^	B (95% CI)
*Univariate models*										
Mode of birth (CS)	≤ 0.01	0.10 (− 0.15 to 0.34)	≤ 0.01	0.06 (− 0.19 to 0.30)	≤ 0.01	− 0.06 (− 0.31 to 0.18)	≤ 0.01	− 0.56 (− 1.46 to 0.33)	≤ 0.01	− 0.17 (− 0.84 to 0.49)
Induction of labor	≤ 0.01	0.02 (− 0.25 to 0.30)	0.02	0.28 (0.01 to 0.55)*	≤ 0.01	0.04 (− 0.24 to 0.31)	≤ 0.01	0.11 (− 0.87 to 1.10)	≤ 0.01	0.47 (− 0.26 to 2.20)
Parity	≤ 0.01	− 0.14 (− 0.35 to 0.08)	≤ 0.01	− 0.14 (− 0.35 to 0.08)	≤ 0.01	− 0.03 (− 0.24 to 0.19)	≤ 0.01	0.17 (− 0.60 to 0.95)	≤ 0.01	0.07 (− 0.51 to 0.65)
Gestational age at birth	≤ 0.01	− 0.03 (− 0.14 to 0.07)	≤ 0.01	0.08 (− 0.02 to 0.19)	≤ 0.01	− 0.05 (− 0.15 to 0.06)	≤ 0.01	0.01 (− 0.37 to 0.38)	≤ 0.01	− 0.02 (− 0.31 to 0.26)
Child sex (boy)	≤ 0.01	− 0.10 (− 0.31 to 0.12)	≤ 0.01	0.14 (− 0.07 to 0.35)	≤ 0.01	0.07 (− 0.15 to 0.29)	≤ 0.01	0.06 (− 0.72 to 0.83)	≤ 0.01	− 0.43 (− 1.00 to 0.15)
Maternal depressive symptoms (BDI-II)^b^	≤ 0.01	0.0 (− 0.02 to 0.020)	0.03	0.02 (0.01 to 0.04)*	≤ 0.01	− 0.01 (− 0.03 to 0.01)	0.04	0.10 (0.03 to 0.17)*	0.14	0.15 (0.10 to 0.20)*
*Multivariate model total sample*										
Mode of birth (CS)	≤ 0.01	0.06 (− 0.21 to 0.32)	0.05	0.04 (− 0.22 to 0.30)	≤ 0.01	− 0.05 (− 0.32 to 0.21)	0.05	− 0.40 (− 1.34 to 0.53)	0.16	− 0.24 (− 0.90 to 0.24)
Induction of labor		0.09 (− 0.23 to 0.41)		0.18 (− 0.12 to 0.49)		− 0.02 (− 0.34 to 0.29)		− 0.47 (0.40 to 1.57)*		0.49 (− 0.29 to 1.27)
Parity		− 0.15 (− 0.39 to 0.09)		− 0.15 (− 0.38 to 0.08)		− 0.01 (− 0.24 to 0.23)		0.21 (− 0.62 to 1.03)		− 0.08 (− 0.67 to 0.50)
Gestational age at birth		− 0.03 (− 0.15 to 0.08)		0.05 (− 0.06 to 0.16)		− 0.06 (− 0.17 to 0.06)		− 0.11 (− 0.51 to 0.29)		− 0.10 (− 0.39 to 0.19)
Child sex (boy)		− 0.06 (− 0.29 to 0.18)		0.08 (− 0.14 to 0.31)		0.03 (− 0.20 to 0.26)		− 0.16 (− 0.96 to 0.65)		− 0.38 (− 0.96 to 0.20)
Maternal depressive symptoms (BDI-II)^b^		0.00 (− 0.02 to 0.002)		0.03 (0.01 to 0.04)*		− 0.01 (− 0.03 to 0.01)		0.10 (0.03 to 0.17)*		0.15 (0.10 to 0.20)*
*Multivariate model girls*										
Mode of birth (CS)	0.04	0.05 (− 035 to 0.45)	0.14	0.06 (− 0.29 to 0.42)	0.03	**− **0.16 (− 0.51 to 0.19)	0.07	− 0.36 (− 1.68 to 0.96)	0.24	− 0.85 (− 1.84 to 0.15)
Induction of labor		0.38 (− 0.09 to 0.84)		0.46 (0.05 to 0.87)*		0.08 (− 0.33 to 0.49)		− 0.69 (− 2.22 to 0.84)		0.55 (− 0.56 to 1.66)
Parity		− 0.24 (− 0.59 to 0.11)		− 0.07 (− 0.38 to 0.23)		− 0.14 (− 0.44 to 0.17)		0.38 (− 0.75 to 1.51)		0.42 (− 0.42 to 1.26)
Gestational age at birth		− 0.04 (− 0.22 to 0.14)		0.12 (− 0.04 to 0.28)		− 0.09 (− 0.24 to 0.07)		− 0.11 (− 0.71 to 0.48)		0.07 (− 0.37 to 0.50)
Maternal depressive symptoms (BDI-II)^b^		0.01 (− 0.02 to 0.04)		0.03 (0.01 to 0.06)*		− 0.01 (− 0.04 to 0.01)		0.10 (0.01 to 0.20)*		0.16 (0.09 to 0.23)*
*Multivariate model boys*										
Mode of birth (CS)	0.02	0.08 (− 0.29 to 0.45)	0.03	0.02 (− 0.36 to 0.40)	≤ 0.01	0.02 (− 0.38 to 0.43)	0.04	− 0.44 (− 1.82 to 0.94)	0.13	0.34 (− 0.55 to 1.23)
Induction of labor		− 0.24 (− 0.69 to 0.24)		− 0.16 (− 0.62 to 0.30)		− 0.11 (− 0.60 to 0.38)		− 0.24 (− 1.88 to 1.40)		0.39 (− 0.70 to 1.49)
Parity		− 0.05 (− 0.39 to 0.28)		− 0.23 (− 0.57 to 0.11)		0.14 (− 0.23 to 0.50)		0.03 (− 1.22 to 1.27)		− 0.68 (− 1.49 to 0.98)
Gestational age at birth		− 0.04 (− 0.20 to 0.12)		− 0.02 (− 0.18 to 0.14)		− 0.03 (− 0.21 to 0.14)		− 0.10 (− 0.01 to 0.47)		− 0.25 (− 0.64 to 0.14)
Maternal depressive symptoms (BDI-II)^b^		− 0.01 (− 0.04 to 0.02)		0.01 (− 0.02 to 0.04)		0.00 (− 0.03 to 0.03)		0.10 (− 0.01 to 0.21)		0.12 (0.04 to 0.19)*

*p ≤ 0.05

^a^Beta coefficient in the linear regression analyses

^b^Continuous score of the Beck Depression Inventory (BDI-II)

Maternal depressive symptoms (BDI-II score) as reported when the child was 4 years of age occurred most frequently as a significant covariate in the multivariate models. Maternal depressive symptoms were significantly associated with Gross Motor and Personal-Social domains (ASQ) (Table 3), Negative Affectivity (CBQ-VSF) and both Internalizing and Externalizing Problems (SDQ) (Table 4). The R^2^ of the multivariate regression models ranged from 1 to 24%. The highest percentage of explained variance was observed in the multivariate linear regression model assessing the association between CS and Internalizing Problems (SDQ) in girls (Table 4).

## Discussion

The aim of this study was to investigate associations between Cesarean section (CS) and child behavioral and developmental outcomes in four-year-olds born healthy to mothers with no serious pregnancy complications. In analyses adjusted for parity, gestational age, child’s sex, induction of labor, and maternal depressive symptoms, we found a small but significant association suggesting that children born via CS demonstrated better problem solving abilities than children who had been born vaginally. Stratifying by child sex indicated that the effects of CS on problem solving were limited to boys. Girls, on the other hand, scored worse in the Gross Motor domain if they were born via CS rather than vaginally, and this effect was not observed in boys. No associations were found between mode of birth and other developmental domains, child temperament or behavioral difficulties.

Our results indicating that CS is associated with better scores in the problem solving domain among boys contradict our hypothesis as we expected that CS would be associated with less optimal outcomes. One explanation for this finding could be that males are more vulnerable to pregnancy and childbirth process. For instance, boys were found to have elevated risk of stillbirth [51] and intrapartum complications, which might be, in part, related to their heavier weight and greater head circumference at birth compared to girls [52]. It is possible that CS diminished risks related to vaginal birth in boys more frequently in comparison to girls, who face fewer risks of vaginal delivery. However, our results need to be considered with caution as research on the effects of CS on child neurodevelopment is still at a very early stage. Moreover, previous studies focusing on the association between CS and cognitive development observed negative effects [20–22], and only one study reported sex-specific associations [22]. Using the same questionnaire (ASQ) as in the present study, Al Khalaf et al. [22] found that girls born via emergency CS were more likely to demonstrate delays in the Problem Solving domain compared to their vaginally born counterparts. They, however, found no such an effect in boys. While Al-Khalaf [22] analyzed the effects of CS on cognitive outcomes in nine-month olds, other authors reporting negative effects of CS on child cognition included children aged 4–9 [21] and 6 years [20]. Of note, our study differs from previously mentioned studies in terms of the sample characteristics. Whereas our study was based on a low-risk sample of healthy mothers and children excluding women with serious pregnancy and perinatal health complications, thus limiting the range of indications for CS, Al Khalaf et al. [22] and Polidano et al. [21] used more medically diverse samples and González-Valenzuela et al. [20] included only twins.

Another finding of the present study is that girls scored slightly worse in terms of their gross motor skills if born by CS instead of vaginally, which was not true for boys. Al Khalaf et al. [22] also found a sex-specific result in this domain, but the higher risk of a gross motor delay concerned girls (and not boys) who were born through instrumental vaginal birth. Both elective and emergency CS were found to be a risk factor for delay in gross motor development in the whole sample [22]. The comparability of the current study and that conducted by Al Khalaf et al. [22] is limited, however, as Al Khalaf et al. assessed motor development in infants, while our study used a sample of pre-school aged children. Assessing the association between CS and mild motor disability in 10-year-old children, Hands et al. [53] found negative effects of CS in boys only. However, Grace et al. [23] reported that both girls and boys were at a higher risk for worse outcomes in motor development in childhood and adolescence if born through either elective or non-elective CS compared to vaginal birth. The inconsistencies in the existing studies may be due to several factors including timing of measurement (i.e. the age of child assessment), method of measurement (parent report or direct assessment), or separation of CS variable into subtypes (elective, non-elective).

We observed no association between CS and emotional or behavioral problems in preschool children. This is in agreement with other studies reporting no differences between either planned or emergency CS and vaginal birth in those domains for children aged 3 [22] and 7 [26]. However, previous studies focusing specifically on maternally requested CS have yielded conflicting results: Li et al. [30] concluded that preschool children born via CS are at a lower risk of externalizing problems while Kelmanson [39] observed a higher risk of internalizing problems in CS-born children at age 5. A recent large study by Huang et al. [37], using the SDQ, supported the results reported by Kelmanson [39], concluding that children born to mothers who requested CS suffered from internalizing problems more frequently compared to vaginally born children, provided that CS was performed prior to the 39th gestational week. Since maternally requested CS has been found to be associated with higher maternal psychological vulnerability, such as severe fear of childbirth or trait anxiety [54], the higher rate of internalizing problems in their children might be related to genetic and family environment factors rather than mode of birth.

The present study has several strengths. We used prospectively collected data to examine the effect of CS on a broad range of developmental domains. Data regarding labor and delivery were extracted from medical records in cooperation with maternity hospitals, rather than relying upon maternal recollection of intrapartum medication and interventions. Our analyses controlled for multiple relevant covariates, and our sample of healthy mother–child pairs with satisfactory perinatal outcomes diminished confounding by health complications that could lead to both CS and compromised child outcomes.

However, several limitations to our study need to be considered. First, the child developmental and behavioral outcomes were assessed by maternal report only, not by direct assessment of child development and behavior. Even though the ASQ, CBQ-VSF and SDQ are widely used, valid and reliable tools to assess child development and behavior, it is not clear to what extent they measure child behavior per se versus maternal *perceptions* of the child. Second, although common in longitudinal studies, the attrition rate was relatively high, with statistical differences found between women who dropped out of the study and those who did not. The women who were more likely to participate in the follow-up study were older, had higher educational status, and were living with a spouse, which might limit generalizability of our findings to a population of women with higher socioeconomic status. Similarly, the exclusion criteria set to eliminate confounding by indication for CS resulted in a sample consisting of a relatively narrow population of healthy mother–child pairs, limiting generalization to mothers at higher risk for adverse outcomes. Also, the sample size was relatively small, which may have limited statistical power, especially when it comes to the analyses stratified by child sex. It is thus possible that some associations detectable in a larger sample were not identified in our study. Moreover, the relatively small sample size precluded us from assessing the effects of planned and emergency CS separately, which may have obscured their differential effects. The variance explained by some models was relatively low, indicating that other unobserved factors explain child outcomes. For example, we did not include maternal parenting competences or breastfeeding status in our models, although those variables might play a role in the association between CS and child outcomes.

Previous investigations have suggested possible adverse consequences of CS on child neurodevelopment, yet our study largely failed to support this association. Results of this study indicate some sex-specific effects of CS, with boys scoring better in the cognitive domain and girls worse in the gross motor domain. Nevertheless, more research is needed before any strong conclusions can be made, preferably using population-based samples and objective measures to assess child outcomes, distinguishing between planned and emergency CS, controlling the analyses for relevant confounds including indications for CS, and conducting sensitivity analyses for high- and low-risk populations. Also, research that might help to identify the mechanisms underlying any association between CS and child outcomes, examining both biological (cortisol response, gut microbiota composition, DNA methylation analysis) and psychosocial (maternal parenting competences, bonding) pathways, is warranted. As there is not sufficient research to draw a final conclusion regarding the effects of CS, a “precautionary principle” approach is encouraged, weighing the benefits and potential risks of the surgical birth intervention for each mother and child.

## Summary

The aim of this study was to investigate associations between Cesarean section (CS) and behavioral and developmental outcomes in 4-year-olds born healthy to mothers with no serious pregnancy complications. Although several previous studies suggested that birth via CS might be associated with adverse developmental and behavioral outcomes, our study largely failed to support this association. We found a small but significant association indicating that children born via CS show better problem solving abilities than children who had been born vaginally. Moreover, stratifying by child sex revealed that the effects of CS on problem solving were limited to boys. Girls, on the other hand, scored worse in the Gross Motor domain if they were born via CS rather than vaginally, and this effect was not observed in boys. We found no evidence that CS may affect other developmental domains, child temperament or behavioral difficulties. More research is needed to substantiate these findings, preferably using population-based samples and objective measures to assess child outcomes, distinguishing between planned and emergency CS, controlling the analyses for relevant confounds including indications for CS, and conducting sensitivity analyses for high- and low-risk populations.

## Electronic supplementary material

Below is the link to the electronic supplementary material.

Supplementary file1 (DOCX 150 kb)
