# Supplementary material for: Associations Between Mode of Birth and Neuropsychological Development in Children Aged 4 Years: Results from a Birth Cohort Study
Source: Child Psychiatry Hum Dev. 2020 Oct 31;52(6):1094–105. doi: 10.1007/s10578-020-01084-4 (PMC8528797; doi:10.1007/s10578-020-01084-4)
Supplement: Supplementary file 1 — Supplementary file1 (DOCX 150 kb) [file 10578_2020_1084_MOESM1_ESM.docx]

**Figure S1 Flow chart of participants**


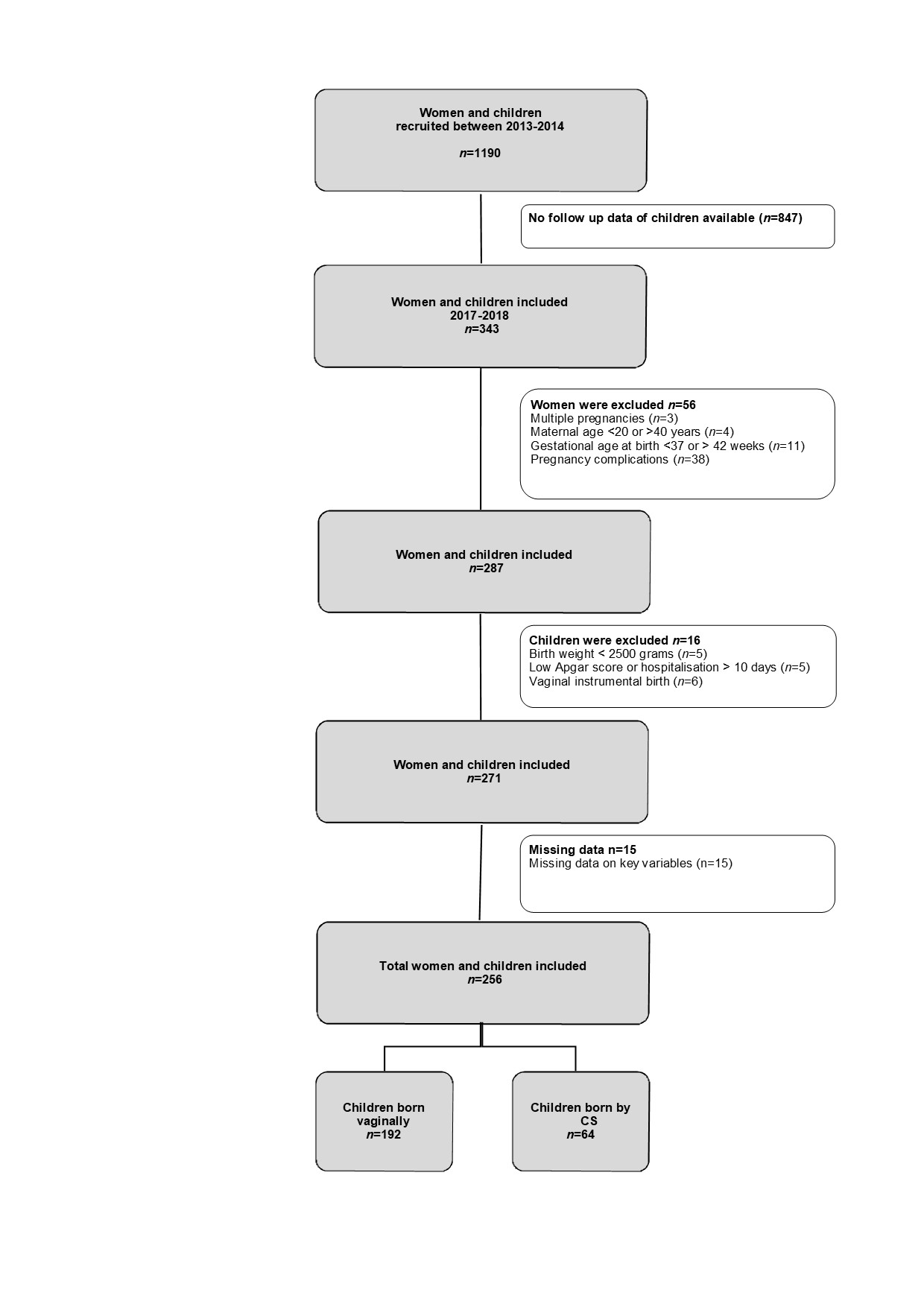


**Table S1 Comparison of women who were included in the final sample and who dropped-out of the study or were excluded based on exclusion criteria; data derived from five maternity wards of non-academic hospitals located in the Vysočina Region, Czech Republic, between 2013 and 2014**

|  | | **TOTAL INCLUDED POPULATION**  **N (%)** | **TOTAL EXCLUDED POPULATION**  **N (%)** | **Statistical differences between included vs. excluded**  ***p*-value** |
| --- | --- | --- | --- | --- |
|  | | ***N*=256** | ***N*=934** |  |
| **MATERNAL CHARACTERISTICS** | |  |  |  |
| **Age** | |  |  | ≤0.001 |
|  | 20-24 years | 12 (4.7) | 84 (9.0) |  |
|  | 25-29 years | 79 (30.9) | 337 (36.1) |  |
|  | 30-34 years | 119 (46.5) | 309 (33.1) |  |
|  | 35-40 years | 46 (18) | 152 (16.3) |  |
|  | Missing |  | 52 (5.6) |  |
| **Educational level** | |  |  | ≤0.001 |
|  | Vocational | 20 (7.8) | 103 (11.0) |  |
|  | Secondary | 122 (47.7) | 484 (51.8) |  |
|  | University | 111 (43.4) | 268 (28.7) |  |
|  | Missing | 3 (1.2) | 79 (8.5) |  |
| **Marital status at baseline** | |  |  | ≤0.001 |
|  | Married | 194 (75.8) | 575 (61.6) |  |
|  | Single/divorced | 61 (23.8) | 315 (33.7) |  |
|  | Missing | 1 (0.4) | 44 (4.7) |  |
| **Parity** | |  |  | 0.82 |
|  | Nulliparous | 121 (47.3) | 434 (46.5) |  |
|  | Multiparous | 135 (52.7) | 500 (53.5) |  |

**Table S2 Mean scores on the measures of developmental (Ages and Stages Questionnaire) and behavioral (Children’s Behavior Questionnaire and Strength and Difficulties Questionnaire) outcomes stratified by the type of CS**

|  | | **Score**  **CESAREAN SECTION** | **Score**  **ELECTIVE CS**  **n=28** | **Score**  **EMERGENCY CS**  **n=36** | **Statistical differences among groups differentiated by type of CS** |
| --- | --- | --- | --- | --- | --- |
| **Developmental outcomes** | | **Mean (SD) ^a^** | **Mean (SD) ^a^** | **Mean (SD) ^a^** | **P-value** |
|  | ASQ Communication | 55.2 (6.9) | 54.0 (7.9) | 56.3 (5.8) | 0.23 |
|  | ASQ Fine motor | 47.1 (12.4) | 44.6 (13.5) | 49.4 (11.0) | 0.17 |
|  | ASQ Gross motor | 51.7 (9.5) | 51.3 (10.8) | 52.0 (8.3) | 0.81 |
|  | ASQ Problem solving | 55.4 (6.2) | 56.3 (4.3) | 54.6 (7.4) | 0.35 |
|  | ASQ Personal-Social | 52.4 (7.7) | 54.1 (6.7) | 51.1 (8.3) | 0.17 |
| **Behavioral outcomes** | |  |  |  |  |
|  | CBQ Effortful control | 5.5 (0.9) | 5.4 (1.0) | 5.6 (0.8) | 0.44 |
|  | CBQ Negative affectivity | 3.9 (0.9) | 3.7 (1.0) | 4.2 (0.9) | **0.04** |
|  | CBQ Surgency | 4.3 (0.9) | 4.5 (0.9) | 4.2 0.8) | 0.09 |
|  | SDQ Externalizing problems | 10.0 (3.4) | 10.1 (3.7) | 9.9 (3.1) | 0.79 |
|  | SDQ Internalizing problems | 6.5 (2.5) | 6.2 (1.9) | 6.7 (2.8) | 0.44 |

^a^ Standard deviation
